# A conversation with Dr. Chi-yuan Hsu, Professor and Division Chief, University of California, San Francisco

**DOI:** 10.1017/cts.2022.463

**Published:** 2023-01-03

**Authors:** Kathy Siranosian

**Affiliations:** Clinical Research Forum, Washington, DC, USA

## Top 10 Clinical Research Achievement Awards Q & A

This article is part of a series of interviews with recipients of Clinical Research Forum’s Top 10 Clinical Research Achievement Awards. This article is with Dr Chi-yuan Hsu, MD, MSc, Professor and Division Chief, University of California, San Francisco. Dr Hsu looked at participants who were enrolled in the Chronic Renal Insufficiency Cohort (CRIC) study. The goal of his study *Race, Genetic Ancestry, and Estimating Kidney Function in Chronic Kidney Disease* was to determine if researchers could estimate glomerular filtration rate (GFR) without considering race.^1^ This study received a 2022 Distinguished Clinical Research Achievement Award. The award recognizes top studies showing creativity, innovation, or a novel approach that demonstrates an immediate impact on the health and well-being of patients. *The interview has been edited for length and clarity.*

## When did you first become interested in clinical research?

My father was a history professor whom I admired a lot so I was always interested in research. I worked in a basic science lab when I was an undergraduate and again in medical school because at the time that was the expected path. But the work didn't really excite me. Then, for my medical school honors thesis, I did a history of medicine project. It was a completely different type of research, and it just clicked with me. I really enjoyed it. That’s when I realized I could like research and I started thinking about how I wanted to do it.

## So at the point, you began charting a new path?

Well, when I was applying for a nephrology fellowship, I still wrote in my essays that I wanted to work in a basic science lab. Again, that’s what the expectation was. I was in Boston and it was the mid-1990s. Clinical research was still very new, and 90% of the Harvard teaching hospital nephrology faculty were doing basic science research. But during the interview process, I began feeling like I wasn't being honest with myself. I knew that what I really wanted to do was clinical research, and eventually, I just started talking about my preference in my interviews. That turned out to be a very pivotal decision. There weren't many role models back then, but I was very fortunate that there were two people at Harvard who were doing clinical research, and they ended up being very influential in my career. Clinical research was a much better fit for me, and I feel it offers much better synergy with being a physician.

## How so?

When I see problems clinically, it inspires me to do research. Then, being a researcher allows me to understand the clinical issues better, which in turn makes me better physician. It’s a very good synergy.

## What was the inspiration for your research about using genetic ancestry, rather than race, to estimate kidney function in chronic kidney disease?

After the George Floyd murder, there was a racial reckoning across the country — and across medicine. In nephrology, people had questions about the two most popular equations we use to assess kidney function, which both require the use of a race co-efficient. These questions had been brewing for quite some time, but they never entered the mainstream, and there had been no serious efforts to address them. Our study showed that the use of serum cystatin C rather than serum creatinine to estimate glomerular filtration rate (GFR) produced estimates of similar validity while eliminating the negative consequences of race-based approaches.

## In what other ways have you seen clinical research change over the course of your career?

One of the biggest changes is that there’s so much more of it being done. Right now, for research-oriented trainees entering our renal fellowship program, most, more than 80% I’d say, are choosing clinical research.

## And why do you think that is?

I think that as a physician, it has become quite difficult to do bench research. That’s because in many ways, bench research has become less connected to clinical medicine. In the past, it was more related to physiology, but now it’s quite specialized. Plus, there is competition from full-time PhD researchers. I really admire people who continue to be successful bench research physician-scientists. By contrast, with clinical research, there’s a direct connection to what’s happening every day with your patients, so it’s easier and can be a more natural fit for physicians.

## What advice do you have for those beginning their career in clinical research?

It’s great career. As physicians, we get to help people — and that’s always a great thing. However, you do have to understand that practicing medicine is a craft. It’s a skilled profession and not necessarily a creative endeavor. The research is what allows you to be creative. Of course, research can be pretty frustrating when things don't work and sometime peer review can be petty. But as a clinical researcher, you step away from the research and go see a patient, and that’s what keeps me grounded. I can see a patient in a clinic or in the hospital and know immediately that I’m helping someone vs. having your paper or grant rejected. It’s having both — the clinical practice and the research — that is so nice.

The second thing I would say is that while research gives you the opportunity to be creative, you can only succeed if you hit the sweet spot. It’s like a Venn diagram. First, there are the things that you want to understand. Then, there are the questions that the world thinks are important. After determining where those two overlap, you have to figure out what’s doable within a reasonable time frame, what’s financially viable, what fits with where you are in your career, and all sorts of other factors. Finding combinations that work is key. That’s the reality of how you get funding and ultimately get published. When you hit the sweet spot and the research pans out — that’s the really fun part. The idea of science is to ask interesting questions and find creative ways that no one else has thought of to answer them. You could do science in a very workmanship way, but that’s just not as interesting or as gratifying.

## How do you continue to maintain that creative perspective?

I’m very much interested in getting answers to the questions I ask. That alone is very motivating. I’m very curious, and from when I wake up to when I go to sleep, I want to work toward finding answers to questions I posed. I suppose I’m inspired by many things. There are so many interesting questions in clinical medicine if one just looks around carefully. It’s great to be able to do science and be able to answer some subset of them — plus, you get to help people and get paid to do it. What could be better?

